# Color-Shape Associations in Deaf and Hearing People

**DOI:** 10.3389/fpsyg.2016.00355

**Published:** 2016-03-15

**Authors:** Na Chen, Kanji Tanaka, Miki Namatame, Katsumi Watanabe

**Affiliations:** ^1^Research Center for Advanced Science and Technology, The University of TokyoTokyo, Japan; ^2^Faculty of Science and Engineering, Waseda UniversityTokyo, Japan; ^3^Department of Synthetic Design, Tsukuba University of TechnologyTsukuba, Japan

**Keywords:** color-shape association, semantic information, implicit association test, deaf

## Abstract

Studies have contended that neurotypical Japanese individuals exhibit consistent color-shape associations (red-circle, yellow-triangle, and blue-square) and those color-shape associations could be constructed by common semantic information between colors and shapes through learning and/or language experiences. Here, we conducted two experiments using a direct questionnaire survey and an indirect behavioral test (Implicit Association Test), to examine whether the construction of color-shape associations entailed phonological information by comparing color-shape associations in deaf and hearing participants. The results of the direct questionnaire showed that deaf and hearing participants had similar patterns of color-shape associations (red-circle, yellow-triangle, and blue-square). However, deaf participants failed to show any facilitated processing of congruent pairs in the IAT tasks as hearing participants did. The present results suggest that color-shape associations in deaf participants may not be strong enough to be proved by the indirect behavior tasks and relatively weaker in comparison to hearing participants. Thus, phonological information likely plays a role in the construction of color-shape associations.

## Introduction

Kandinsky (1912/1994, 1947), renowned abstract painter, proposed a correspondence theory between shapes and colors that state that circle, square, and triangle are associated with blue, red, and yellow, respectively. Kandinsky’s correspondence theory became influential in art and design theory ever since it was proposed, but, researchers and artists showed different patterns of color-shape associations (Lupton and Miller, 1993; Jacobsen, 2002; Jacobsen and Wolsdorff, 2007; Dumitrescu, 2011; Kharkhurin, 2012; Albertazzi et al., 2013; Chen et al., 2015a,b). For example, Jacobsen (2002) used a modified version of Kandinsky’s questionnaire and found that German participants tended to associate red with triangle, blue with square, and yellow with circle. Dumitrescu (2011) asked American participants to rate the appropriateness of colored-shapes (both two-dimensional and three-dimensional shapes) and found associations between circle or sphere with red, square or cube with blue, and tetrahedron with yellow. Albertazzi et al. (2013) asked Italian participants to choose which color best matched various geometric shapes and found that Italian participants associated circle and square with red, triangle with yellow. Most recently, Chen et al. (2015a) adopted Albertazzi et al. (2013) experimental procedure and examined color-shape associations in Japanese participants. They asked participants to choose the color that best matched a shape (one of the basic 2D and 3D geometric shapes) from a color wheel containing 40 hue colors (from Natural Color System). They reported that Japanese participants systematically associated shapes with colors; rounded shapes (i.e., circles) are matched best with red colors, sharp angular shapes (i.e., triangles) are matched best with yellow colors, and squared shapes (i.e., squares) are matched best with blue colors. These reported color-shape associations are, to some extent, shared in some populations (e.g., Italian and Japanese). Previous studies suggested that color-shape associations were likely influenced by everyday knowledge (e.g., a yellowed triangle resembles the pyramid in Egypt; Jacobsen, 2002), and/or the common semantic information underlying colors and shapes (warm/cold or light/dark in Albertazzi et al., 2013; warm/cold in Chen et al., 2015a).

In addition to the aforementioned explicit tests, some studies used the indirect implicit method to study color-shape associations (Kharkhurin, 2012; Holmes and Zanker, 2013; Makin and Wuerger, 2013). For example, Makin and Wuerger (2013) examined Kandinsky’s color-shape associations by an indirect behavioral method: the Implicit Association Test (IAT). The IAT is designed to reveal people’s unconscious associations between different stimulus dimensions by response times (RTs; Greenwald et al., 1998; Nosek et al., 2007). Their results showed little support for Kandinsky’s correspondence theory, except only a marginal effect for one pair of combinations (square-red and triangle-yellow associations) among the three combinations they tested (the other two combinations: circle-blue and square-red, circle-blue and triangle-yellow associations). Then, Chen et al. (2015b) initially verified whether those Japanese color-shape associations (e.g., circle-red, triangle-yellow, square-blue) could be measured by the IAT. They found that RTs were significantly faster when the visual stimuli of circle-red, triangle-yellow, and square-blue combinations were mapped onto the same response key, rather than different key combinations. Taken together with the results in explicit experimental procedure (Chen et al., 2015a), they suggested that color-shape associations (e.g., circle-red, triangle-yellow, square-blue) were encoded by Japanese participants and could be measured by both direct and indirect methods.

Studies have suggested that humans show a tendency to spontaneously match distinct features and dimensions of experience across different or within sensory modalities, known as crossmodal correspondence (Spence, 2011). For example, people tend to match high-pitched sounds with high spatial locations, bright colors, sharp angular shapes, small sizes, and lighter weights as compared with low-pitched sounds (Ben-Artzi and Marks, 1995; Martino and Marks, 1999; Collier and Hubbard, 2001; Eitan and Timmers, 2010; Spence, 2011). Spence (2011) suggested that crossmodal correspondence could be characterized into three different groups: statistical, structural, and semantically mediated correspondences, which have different developmental trajectories and consequences for human perception and behavior. First, structural correspondences may occur because of neural connections that are present at birth, reflecting a common basis for coding stimuli that share features such as magnitude or intensity (e.g., loudness-brightness association). Second, statistical correspondences may occur through perceptual learning following repeated exposure to co-occurrences in the natural environment (e.g., pitch-elevation association). Third, semantically mediated correspondences may stem from the common semantic meaning or the use of common lexical terms to describe stimuli in different sensory channels (e.g., the words “high” and “low” are used to describe contrasting levels of pitch, spatial elevation, and spatial frequency, which lead to the correspondences among them).

Color and shape are the two basic visual features; the non-arbitrary association between color and shape indicates that correspondence could happen within modality. Those color-shape associations could be explained by common semantic information conveyed by colors and shapes (Chen et al., 2015a), such as the warm (cold) colors tended to be associated with warm (cold) shapes. Martino and Marks (1999) suggested a semantic coding hypothesis to interpret the crossmodal correspondences emerged only after language acquisition (Marks, 1984, 1987; Smith and Sera, 1992). They posited that crossmodal correspondences arise from coding perceptual stimuli into semantic representations, reflecting interactions between linguistic and semantic meanings. Hence, the construction of semantic sensory correspondence could be influenced by language learning experience.

Asano and Yokosawa (2011, 2012) found phonological information appears to influence the grapheme-color associations. They asked grapheme-color synesthesia people to choose colors for the Japanese letter of Hiragana and their Katakana counterparts, representing the same set of syllables but with different visual features. The results showed that the color choices were remarkably consistent for Hiragana and Katakana, which suggested that the letters’ sound might modulate those intra-visual grapheme-color associations. Therefore, we thought that phonological information might also play a part in the construction of color-shape associations.

In the present study, we compared color-shape associations in hearing and deaf participants. If the same semantic sensory correspondence (i.e., the common semantic information underlying properties of sensory features) occurred between colors and shapes, deaf people would likely demonstrate color-shape associations similar to those of hearing people. For example, both the color “red” and the shape “circle” could convey “warmth” information in deaf people, so as to lead to the “circle-red” association. However, due to limited hearing experience, deaf people develop different language systems from hearing (Furth, 1966), which may influence how visual features are encoded semantically. Thus, hearing and deaf participants may have different color-shape associations or have quantitatively different magnitudes of color-shape associations.

In order to compare color-shape associations in deaf and hearing people, we used both direct and indirect experimental methods. In Experiment 1, we used an explicit matching questionnaire survey to directly examine deaf and hearing people’s color-shape associations. Based on the previous study (Chen et al., 2015a), a circle is significantly associated with red, a triangle is associated with yellow, and a square is associated with blue or green colors, thus we chose these three shapes and four colors. In Experiment 2, we implemented an indirect behavioral method using the IAT to measure the strength of color-shape associations in deaf people, and compared their performance with that of hearing people (Chen et al., 2015b).

## Experiment 1

### Method

#### Participants

Ninety-one Japanese college students from Tsukuba University of Technology whose hearing threshold level was over 60 db participated in this survey (32 women, mean age = 21.2, *SD* = 1.3). Hereafter, this group is referred to as deaf participants. The deaf participants were congenitally deaf using Japanese Sign Language (JSL). All participants reported to have normal or corrected to normal visual acuity and normal color vision. This experiment, as well as the subsequent experiment, was approved by the institutional review board of The National University Corporation of Tsukuba University of Technology. Ninety-five hearing Japanese college students also took part in this survey (41 women, mean age = 21.6, *SD* = 2.9) as a control group, they reported to have normal or corrected to normal visual acuity and normal color vision. The experiment with hearing participants was approved by the institutional review board of The University of Tokyo. These experiments were conducted in accordance with the ethical standards in the 1964 Declaration of Helsinki. All of the participants provided informed consent prior to the study.

#### Stimuli and Procedure

**FIGURE 1:**
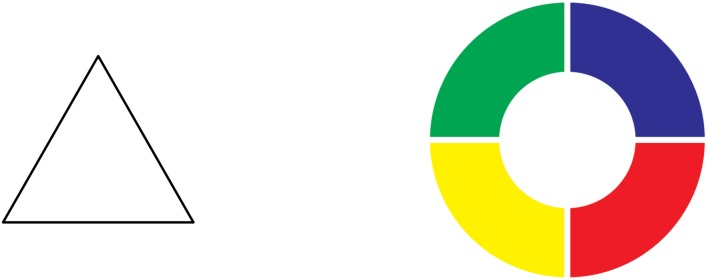
**An example of the questionnaire survey with the triangle shape.** The right color wheel had a small white circle in the center. Four colors (i.e., red, yellow, blue, green) were located around the small center circle.

A questionnaire survey composed of one A4 size page was conducted. Outlines of three basic shapes (i.e., circle, triangle, and square) were presented on the left side of the page. A color wheel filled with four categorical colors (i.e., red, yellow, blue, and green) was presented on the right side of the page (i.e., next to the shapes; **Figure 1**). The order of shapes and the rotation of the color wheel were counterbalanced across participants. The participants were instructed to choose the color that best matched each shape from the four-color wheel. Note that they were allowed to choose the same color for several shapes (e.g., red-circle and red-triangle). The questionnaire survey was conducted individually in a lighted laboratory room in each research institution. A time limit was not set.

#### Data Analysis

We used a log-linear analysis (Christensen, 1997) to examine the relationship between shape, color, and participant group (deaf and hearing), and a chi-square test to reveal specific color-shape associations (Chen et al., 2015a).

### Results and Discussion

#### Frequency of Color Choice for Shapes

We first calculated the frequencies of color choice for each shape for deaf and hearing participants (**Table 1**; Supplementary Tables S1 and S2). In order to reveal the color choices of shapes in the two groups of participants, a log-linear analysis was used to examine the relationships between color, shape, and group. We performed a three-way log-linear analysis: shape (3) × color (4) × group (2), for the accumulated frequencies of color choice for shapes in both deaf and hearing participants. The results indicate that main effect for group [χ(1)^2^ = 0.26, *p* = 0.61], interactions between group and color [χ(3)^2^ = 1.99, *p* = 0.57], and interactions between group, color, and shape [χ(6)^2^ = 12.04, *p* = 0.06] were not statistically significant, signifying that group had little effect on color-shape associations. Moreover, there was a significant main effect for color [χ(3)^2^ = 8.35, *p* < 0.05], and a significant interaction between shape and color [χ(6)^2^ = 252.61, *p* < 0.001], suggesting that color choices for shapes were not random. Taken together, deaf and hearing participants probably have a similar pattern of color-shape associations when assessed in the explicit questionnaire survey.

**Table 1:** Frequency (%) of colors for shapes in hearing and deaf participants.

		Red	Yellow	Blue	Green
Circle	Hearing	**58 (61.05%)**	23 (24.21%)	7 (7.37%)	7 (7.37%)
	Deaf	**59 (64.84%)**	8 (8.79%)	15 (16.48%)	9 (9.89%)
Triangle	Hearing	18 (18.95%)	**45 (47.37%)**	16 (16.84%)	16 (16.84%)
	Deaf	19 (20.88%)	**42 (46.15%)**	9 (9.89%)	21 (23.08%)
Square	Hearing	7 (7.37%)	9 (9.47%)	**45 (47.37%)**	**34 (35.79%)**
	Deaf	5 (5.49%)	11 (12.09%)	**44 (48.35%)**	**31 (34.07%)**

*Cells in bold indicate a significant association between color and shape, *p* < 0.05.*

**FIGURE 2:**
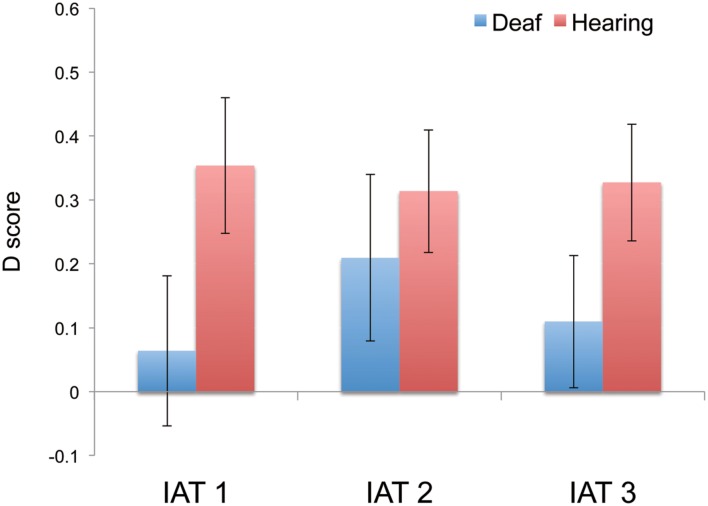
**Mean D scores of deaf and hearing participants in the three IAT tasks.** The data of the hearing participants was referred from Chen et al. (2015b). Error bars represent the standard errors of the mean.

In the next analysis, we combined color choices for shapes by deaf and hearing participants. *Post hoc* chi-square tests were conducted on the color choices for each shape to examine which color was most frequently chosen for each shape. The results showed significant differences in the color choices for each shape [circle: χ(3)^2^ = 144.97, *p* < 0.01; triangle: χ(3)^2^ = 49.09, *p* < 0.01; square: χ(3)^2^= 86.90, *p* < 0.01]. Multiple comparisons using Ryan’s method (Hsu, 1996) showed that certain colors were chosen more frequently for certain shapes (*p*s < 0.05; **Figure 2**). For example, reds were chosen more frequently than the other colors for the circle (62.90%), yellows were chosen more frequently for the triangle (46.77%), and blues and greens were chosen more frequently for the square (blue: 47.85%, green: 34.95%). These results showed that both deaf and hearing participants had established circle-red, triangle-yellow, and square-blue/green associations.

Previously, Chen et al. (2015a) instructed hearing participants to choose the hue color that was the best match for a geometric shape from 40-hue color circles (Natural Color System; following Albertazzi et al., 2013). They found that the participants demonstrated systematic associations between shapes and colors. For example, about 34% of the participants matched circles with red colors, 33% matched triangles with yellow colors, and 19% matched squares with blue colors. The present study supported the previous results.

Taken together, we observed that both deaf and hearing participants tended to associate specific colors with specific shapes (i.e., circle-red, triangle-yellow, and square-blue/green). The similar color-shape associations may indicate the same semantic sensory correspondence between colors and shapes in both the hearing and deaf (i.e., the common semantic information underlying properties of sensory features). However, due to a different language system of deaf and hearing (Furth, 1966), their color-shape associations may have quantitatively different magnitudes of color-shape associations (i.e., the strength of color-shape associations). Thus, in Experiment 2, we implemented an indirect behavioral method to further examine whether the strength of color-shape associations in deaf and hearing participants would be different. For this, we adopted the IAT, which has been widely used to examine the strength of unconscious associations (both explicit and implicit) between different dimensions (Greenwald et al., 1998; Nosek et al., 2007; Gattol et al., 2011; Parise and Spence, 2012; Ho et al., 2014). In a typical IAT task, two pairs of visual stimuli are assigned to two different keys. Participants are instructed to press one of the two keys rapidly and accurately for one pair of visual stimuli, and to press the other key for the other pair of visual stimuli. If the two visual stimuli assigned to the same key are associated for a participant (congruent condition), RTs should be shorter than when the two stimuli assigned to the same response key are not associated (incongruent condition). In our previous study (Chen et al., 2015b), we found that color-shape associations of hearing Japanese people (circle-red, triangle-yellow, and square-blue) could be ascertained using the IAT. In Experiment 2, we used the same IAT experiment paradigm as in the previous study (Chen et al., 2015b) to examine the strength of deaf participants’ color-shape associations (i.e., circle-red, triangle-yellow, and square-blue).

## Experiment 2

### Method

#### Participants

Twenty college students from Tsukuba University of Technology, whose hearing threshold level was over 60 db, participated in this experiment (eight women, mean age = 21.1, *SD* = 1.0). Sixteen of the 20 participants also participated in Experiment 1, with a time gap of approximately 3 months between Experiments 1 and 2. All participants reported to have normal or corrected to normal visual acuity and normal color vision. In order to compare task performance between deaf and hearing participants, we referred to the data of the hearing participants from the previous study (Chen et al., 2015b): twenty-four Japanese college students (eight women, mean age = 21.1, *SD* = 1.4) with normal visual acuity and color vision.

#### Apparatus and Stimuli

We used the same IAT paradigm as in the previous study (Chen et al., 2015b). The previous study with hearing participants was conducted in a laboratory at the University of Tokyo, and the present experiment was performed at the Tsukuba University of Technology. Stimuli were presented on a 1280 × 1024 pixel LCD monitor, with a refresh rate of 60 Hz. During the experiment, only one visual stimulus was shown centrally on the screen in each trial. The shape stimuli were drawn in white lines (with a width of 26 mm, 0.03° visual angle) on a gray background (8 cd/m^2^). The circle was 4.8° in diameter, the square was 4.8° (in height) × 4.8° (in width), and the triangle was 4.8° (in height) × 5.8° (in width). The shape stimuli were presented in an upward orientation. The color stimuli comprised three-color patches that were ∼9.5° wide (Gaussian-modulated). The colored Gaussian patches were measured using PR-655 (Photo Research, Chatsworth, CA, USA) and each color was consecutively measured 10 times and averaged. The color information was as follows: yellow: L^∗^ = 47.5, a^∗^ = 3.07, b^∗^ = 59.47; red: L^∗^ = 18.74, a^∗^ = 36.53, b^∗^ = 26.03; blue: L^∗^ = 21.07, a^∗^ = -2.6, b^∗^ = -21.17.

#### Procedure

The experiment paradigm was identical to that of Experiment 2 in the previous study (Chen et al., 2015b). The experiment was carried out in a dimly lit laboratory. Each participant seated in front of the monitor at a distance of about 60 cm. The experiment had three IAT tasks and each task tested two pairs of color-shape links (i.e., IAT 1: circle-red/square-blue; IAT 2: circle-red/triangle-yellow; IAT 3: square-blue/triangle-yellow). In the task, the participants were instructed to discriminate a centrally shown color or shape stimulus by pressing a key (i.e., “z” as a left key with left index finger or “m” as a right key with right index finger). Each task was comprised of eight blocks; the blocks 1, 2, 5, 6 were the training blocks and the blocks 3, 4, 7, 8 were the test blocks. Here, we showed an example of the procedure in IAT 2.

In Block 1, the participants were trained to discriminate a shape stimulus by pressing a key (e.g., the left key for circle and the right key for triangle in an example of IAT 2; **Table 2**). When the target stimulus (a shape or color) was presented in the center of the screen, the cue words also appeared on the top left and right sides of the screen to remind participants which key to press. For example, in Block 1, the words 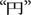 (circle) and 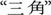 (triangle) appear on the top left and top right sides of the screen according to the response mapping. When the participants pressed a wrong button, the word “ERROR” would appear centrally in red. At the beginning of each block, the participants were appropriately informed of the stimuli, response mapping, and cue words of the upcoming block. An instruction in Block 1 was as follows: “In this block, if you see a circle (○), please press the “z” key with your left index finger. If you see a triangle (△), please press the “m” key with your right index finger. The cue words of 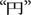 (circle) and 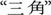 (triangle) will appear at the top left or right sides of the screen to remind you which key to press. Please press the key as accurately and quickly as possible. When you press a wrong key, a red word “ERROR” will appear.” The number of trials in Block 1 was 20.

**Table 2:** Sequence of blocks and response mappings (an example from IAT 2).

Block	Trials	Condition	Left key response	Right key response
1	20	Training 1	Circle	Triangle
2	20	Training 2	Red	Yellow
3	20	Congruent 1	Circle or red	Triangle or yellow
4	40	Congruent 2	Circle or red	Triangle or yellow
5	20	Training 3	Triangle	Circle
6	20	Training 4	Triangle	Circle
7	20	Incongruent 1	Triangle or red	Circle or yellow
8	40	Incongruent 2	Triangle or red	Circle or yellow

In Block 2, the participants were trained to discriminate a color stimulus by pressing a key (e.g., the left key for red, the right key for yellow; **Table 2**). The procedure and instruction in Block 2 were identical to those in Block 1 except a color was used instead of a shape. The number of trials in Block 2 was 20.

In Blocks 3 and 4, response mappings for color and shape stimuli were combined (e.g., left key for circle or red, and right key for triangle or yellow; congruent blocks). The response mapping was in accordance with the color-shape associations found in Experiment 1 (i.e., red-circle, and yellow-triangle, in IAT 2), comprising a congruent block. An instruction in Blocks 3 and 4 was as follows: “If you see a circle (○) or color red, please press key “z” with your left index finger. If you see a triangle (△) or color yellow, please press key “m” with your right index finger. The cue words of 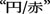 (circle/red) and 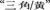 (triangle/yellow) will appear at the top left or right sides of the screen to remind you which key to press.” The number of trials in Blocks 3 and 4 was 20 and 40, respectively.

In Blocks 5 and 6, the participants were instructed to learn an opposite shape-key mapping to that of the previous blocks (e.g., left key for triangle, and right key for circle). The number of trials in Blocks 5 and 6 was 20. The procedure and instruction were identical to those in Block 1 except that the shape-key mapping was opposite.

In Blocks 7 and 8, the participants were instructed to discriminate a color or shape stimulus with response mapping opposite to the observed color-shape associations in Experiment 1, comprising an incongruent block (e.g., left key for triangle or red, and right key for circle or yellow). The number of trials in Blocks 7 and 8 was 20, and 40, respectively. The procedure and instruction were identical to those in Blocks 3 and 4 except that the response mapping was different.

Note that the order of the three IAT tasks was counterbalanced across participants, and in each IAT task, half of the participants performed the congruent blocks first and the other half of the participants performed the incongruent blocks first, and the training blocks were rearranged accordingly. In each block of the IAT task, the order of stimulus presentation was randomized for each participant. **Table 3** shows an example of response mappings in the three IAT tasks. Each IAT task took about 7 min, and the participants rested about 3 min between tasks; thus, the whole experiment took about 30 min.

**Table 3:** Response mappings in the IAT tasks.

	IAT 1		IAT 2		IAT 3	
Blocks	Left key	Right key	Left key	Right key	Left key	Right key
Congruent blocks	Circle	Square	Circle	Triangle	Square	Triangle
	Red	Blue	Red	Yellow	Blue	Yellow
Incongruent blocks	Square	Circle	Triangle	Circle	Triangle	Square
	Red	Blue	Red	Yellow	Blue	Yellow

#### Data analysis

After error trials were excluded, the RTs were used for data analysis. D scores were calculated to reveal the associations between colors and shapes (Greenwald et al., 2003; Nosek et al., 2007; Chen et al., 2015b). D score provides a measure of the RT difference between the congruent and incongruent blocks (3, 4, 7, and 8), as a function of the standard deviation of the distribution. The D scores were calculated as follows. The RTs in the congruent block (Block 3 for those who performed a congruent block first, and Block 7 for those who performed an incongruent block first) were subtracted from those in the incongruent block and the differences were divided by their standard deviation. The same procedure was repeated for the RTs in Blocks 4 and 8. Finally, the mean of the two computed values was defined as D score: D score = 1/2 × [(RT_7or3_ – RT_3or7_) / SD_(3,7)_ + (RT_8or4_ – RT_4or8_)/SD_(4,8)_]. A positive D score value indicates support for the proposed hypothesis of color-shape mappings, and a negative value indicates that the participant displayed opposite associations to the hypothesis. The D score was computed in each IAT task, resulting in three D scores for each participant (See Supplementary Table S3).

We performed one-sample *t*-tests for the obtained D scores and zero with Bonferroni corrections following the analysis procedures used in the previous studies (Nosek et al., 2007; Gattol et al., 2011; Makin and Wuerger, 2013; Chen et al., 2015b). In addition, we compared the RTs in congruent and incongruent conditions in each of the three IAT tasks using the paired *t*-tests with Bonferroni corrections. The RTs should theoretically be shorter in the congruent conditions than in the incongruent conditions if the participants established the hypothesized color-shape associations.

We also compared D scores and RTs of deaf and hearing participants using a linear mixed-effects model (e.g., Baayen et al., 2008; Bates et al., 2012). We used the data of hearing participants in Chen et al. (2015b). Here, we adopted this approach because a linear mixed model allows for variation in effects based on random factors such as participants. In the comparison of D scores, we included group (hearing and deaf), and task (three IAT tasks) as fixed effects and participants as a random effect. In the comparison of RTs, we included group (hearing and deaf), task (three IAT tasks), and congruency (congruent and incongruent conditions) as fixed effects and participants as a random effect.

### Results and Discussions

We analyzed task performance of deaf participants in this session. One participant whose error rate more than 15% was excluded from data analysis. We excluded error trials (4.5%) and trials with RTs longer than 2000 ms (0.2%). All individual-level data including participant information, D score, RT, and error rate are provided in Supplementary Table S1.

#### D Scores

We used one-sample *t*-tests with Bonferroni corrections to compare D scores with zero. The results showed that D scores in the three IAT tasks were normally distributed around zero [IAT 1: *t*(18) = 0.54, adjusted *p* > 0.99, mean D score = 0.06, 95% *CI*[-0.18 0.31]; IAT 2: *t*(18) = 1.61, adjusted *p* = 0.78, mean D score = 0.21, 95% *CI*[-0.06 0.48]; IAT 3: *t*(18) = 1.06, adjusted *p* > 0.99, mean D score = 0.11, 95% *CI*[-0.11 0.33]; **Figure 2**]. In addition, further analysis applying the bootstrap method with 2,000 iterations indicated that IAT 2 revealed a marginal significant difference (*p* = 0.08), while IAT 1 and IAT 3 did not show significant differences (*p*s > 0.1). The confidence interval of the D-score and the results of the bootstrap indicated that the deaf participants might demonstrate a weak circle-red and triangle-yellow association to some extent.

#### Response Time

**FIGURE 3:**
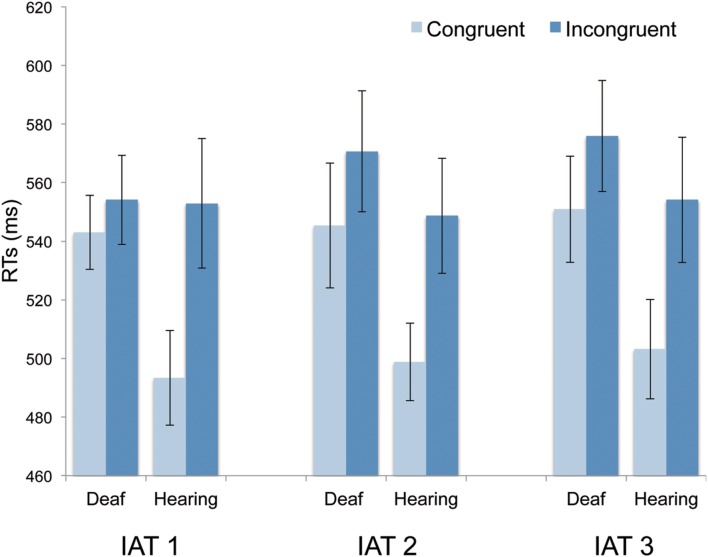
**Mean RTs in congruent and incongruent blocks for deaf and hearing participants in the three IAT tasks.** The data of the hearing participants was referred from Chen et al. (2015b). Error bars represent the standard errors of the mean.

Paired *t*-tests with Bonferroni correction were used to compare the mean RTs in congruent and incongruent conditions. The results did not reveal any significant difference on RTs between congruent and incongruent conditions in all three of the IAT tasks [IAT 1: *t*(18) = 0.74, adjusted *p* > 0.99, Cohens’*d* = 0.35; 543 ms in the congruent condition (*SD* = 54.85) vs. 554 ms in the incongruent condition (*SD* = 66.43); IAT 2: *t*(18) = 1.14, adjusted *p* > 0.99, Cohens’*d* = 0.35; 545 ms (*SD* = 92.84) vs. 571 ms (*SD* = 89.94); IAT 3: *t*(18) = 1.45, adjusted *p* > 0.99, Cohens’*d* = 0.68; 551 ms (*SD* = 79.07) vs. 576 ms (*SD* = 82.66); **Figure 3**]. The average mean RTs across the three IAT tasks in the congruent and incongruent conditions also failed to demonstrate any significant difference [*t*(18) = 1.93, adjusted *p* = 0.42, Cohens’*d* = 0.91; 546 ms (SD = 57.93) vs. 567 ms (*SD* = 65.32)]. Therefore, there was no significant difference on RTs between congruent and incongruent conditions along the three IAT tasks. Taken together, the results of D scores and RTs provided limited evidence for the congruency effect of color-shape associations, which suggests that deaf participants might have weak or limited color-shape associations, as revealed by the IAT.

#### Comparisons of Color-Shape Associations between Hearing and Deaf Participants

Chen et al. (2015b) conducted the same three series of IAT tasks with twenty-four hearing Japanese participants and they found a significant congruency effect of color-shape associations in all three IAT tasks in terms of D-scores and RTs (as shown in **Figures 2** and **3**). Here, we discuss the difference in IAT performance between deaf and hearing participants. As the numbers of deaf and hearing participants were different (19 vs. 24 participants), we used a bootstrap analysis to examine whether the different results from the present and previous studies were due to the different sample number of participants.

We firstly examined whether the re-sampled D-scores in hearing participants (*N* = 24) were larger than zero. The re-sampled D scores with 2,000 bootstrap iterations showed that all three IAT tasks revealed significant differences from zero (all *p*s < 0.01). Next, we examined whether the re-sampled D scores in hearing participants equivalent to the number of deaf participants (*N* = 19) were larger than zero. The re-sampled D scores with 2,000 iterations showed significant positive results across all three IAT tasks (all *p*s < 0.01). These results indicated that even with a smaller sample, hearing participants still demonstrated the significant congruency effect with color-shape associations across the three IAT tasks.

The results of the linear mixed model for D scores did not show significant effects of intercept (*p* = 0.65), group (*p* = 0.21), task (*p* = 0.77), and an interaction between group and task (*p* = 0.73) as fixed effects and of participants (*p* = 0.29) as a random effect. This results indicates that the D scores do not significantly differ between the hearing and deaf participants. However, as the D scores in the three IAT tasks in the hearing participants were significantly larger than zero and those in the deaf were not significantly larger than zero, deaf and hearing participants might have slightly different strength of color-shape associations.

The results of the linear mixed model for RTs showed significant effects of intercept (*p* < 0.001), group (*p* < 0.001), congruency (*p* < 0.001), but did not show significant differences of task (*p* = 0.38), and interactions among group, task, and congruency (*ps* > 0.07). The model also showed a significant effect of participants as a random effect (*p* < 0.001). These results indicated that the RTs were generally shorter in the hearing participants than in the deaf participants and also were shorter in the congruent condition than in the incongruent condition.

## General Discussion

In the present study, we observed that both deaf and hearing participants demonstrated qualitatively similar patterns of color-shape associations using a direct questionnaire survey (e.g., circle-red, triangle-yellow, and square-blue/green), which was consistent with the previous study (Chen et al., 2015a). However, deaf participants showed a limited and less pronounced congruency effect of color-shape associations when the indirect behavioral task (IAT) was used. These results suggest that deaf participants might have a relatively weaker form of color-shape associations compared with hearing participants.

In Experiment 1, we did not observe any significant difference of color-shape associations between deaf and hearing participants in the direct questionnaire survey. Chen et al. (2015a) demonstrated that the common semantic information (e.g., warm/cold) for colors and shapes lead to color-shape associations. It could be possible that deaf participants also may think that circle has a warm feeling and red is warm, and they made the circle-red association as the hearing participants do (Chen et al., 2015a). Fonagy (2001) reported that deaf children with limited hearing experience exhibited the same mappings of proprioceptive, tactile, and motor sensations as children and adults with normal hearing. For example, the words “*k*” and “*r*” are harder than “*l*” (Fonagy, 1963, 2001). Therefore, deaf participants might be able to refer to the common semantic information related with colors and shapes when asked directly, and showed similar pattern of color-shape associations.

In Experiment 2, in the indirect IAT tasks, the participants were required to response quickly and the RTs are used to reveal the unconscious associations and the strength of those associations. The results showed that the RTs were generally slower in deaf participants than in hearing participants. The slower RTs in the deaf participants may depend on types of task. For example, deaf people have been reported to show either similar or worse performance on a variety of tasks as compared with hearing people, such as behavioral control, visual attention, visual search, and processing of central distractors (Parasnis et al., 2003; Quittner et al., 2004; Bavelier et al., 2006). However, the acquisition of sign language has been reported to enhance some high cognitive performance, such as mental rotation, image generation, and short-term memory (Emmorey et al., 1993; Capirci et al., 1998; Boutla et al., 2004). Yang and Tan (2008) used the IAT and found that deaf students have high self-esteem effect as normal hearing students do; the deaf students could perform comparably as normal hearing students in the IATs. Furthermore, previous studies suggested that deaf people tended to be superior in some visual processing, such as in visual motion detection and peripheral distractor processing (Neville and Lawson, 1987; Bosworth and Dobkins, 2002; Bavelier et al., 2006; Bottari et al., 2010). The present results may indicate that the deaf participants are not good at rapidly discriminating a centrally shown stimulus while this issues needs a further examination. That said, the slower RTs in the deaf participants did not pertain to the non-significant difference of the RTs between the congruent and incongruent blocks in deaf participants; deaf participants failed to show any acceleration of RTs with the congruent effect of color-shape associations as the hearing participants did. These results indicate that deaf participants might have limited color-shape associations, which was not strong enough to be proved by the indirect IATs.

Two possibilities might arise to account for the limited color-shape associations in deaf participants: different language development between deaf and hearing and better visual processing in deaf than in hearing. First, according to the semantic coding hypothesis (Martino and Marks, 1999), cross-sensory interactions observed in speeded classification tasks arise after perceptual information is recoded into a common semantic basis. In the present study, for hearing participants, colors and shapes might be recoded into a common semantic basis (e.g., warm/cold), where the color-shape interactions occurred and the congruent pairs (e.g., “warm” based circle-red pair) would accelerate RTs to discriminate stimulus. An occurrence of the congruency effect is mainly comprised of two parts; first, with time and experience, perceptual information was incorporated into language; second, the correspondence arose from the coding and recoding of perceptual information into the semantic basis. Due to a lack of phonological information, deaf people developed a sign language (Furth, 1973; Fonagy, 2001; Tamariz, 2004). The different language development may influence the semantic encoding of perceptual stimuli, which in turn influence the strength of those associations. Previous studies suggested that the correspondence between phonological information and meaning contribute to the overall structure of a language, and phonological information tends to aid form-meaning associations (Tamariz, 2004, 2008; Monaghan et al., 2007). Therefore, phonological information might play a role on the construction of color-shape associations. For example, Chen et al. (2015a) showed that hearing people could use the adjective word “warm” to describe both color red and shape circle, and the red-circle association might be constructed through the common semantic meaning of “warm.” It may be also possible that deaf people could perceive the feeling of “warm” when they see the red or circle, and the red-circle association based on the “warm” perception could exist when directly asked. Several studies suggested that phonological information emphasized the meaning of visual stimuli (Van Orden, 1987; Tan and Perfetti, 1997). Thus, lack of phonological information might influence the profound semantic processing of sensory stimuli, which might lead to the weak form of unconscious color-shape associations in deaf people.

Second, deaf participants might be better at separating visual features (i.e., color and shape) than hearing participants, which leads to the less pronounced congruency effect of color-shape associations on accelerating RTs to discriminate visual stimulus. Color and shape are the two visual features that should be treated independently for efficient coding of a visual scene (unless there are high correlations between them). One prediction from this conjecture is that the interactions between visual modalities (e.g., illusory conjunction, color-motion feature binding, Wolfe and Cave, 1999; Holcombe and Cavanagh, 2001; Robertson, 2003; Wu et al., 2004; Noguchi et al., 2011) would be less pronounced in deaf participants than in hearing participants. However, this possibility requires further empirical investigations.

Here, we would like to explain a limitation of the present study. The aim of the present study was to compare deaf and hearing people’s color-shape associations using both direct and indirect experimental method. In Experiment 2 with the IAT tasks, we recruited 19 deaf participants, and compared them with 24 hearing participants (Chen et al., 2015b). Even though we used the bootstrap analysis method, the present result might be underpowered with the small sample size. Future study might be helpful to examine the deaf people’s IAT performance, and compare with hearing people with a larger sample size.

## Author Contributions

Conceived and designed the experiments: NC, KT, MN, and KW. Performed the experiments: NC, KT, and MN. Analyzed the data: NC and KT. Contributed reagents/materials/analysis tools: NC, KT, MN, and KW. Wrote the paper: NC, KT, MN, and KW.

## Conflict of Interest Statement

The authors declare that the research was conducted in the absence of any commercial or financial relationships that could be construed as a potential conflict of interest.
